# Assessing Kidney Injury Induced by Mercuric Chloride in Guinea Pigs with In Vivo and In Vitro Experiments

**DOI:** 10.3390/ijms24087434

**Published:** 2023-04-18

**Authors:** Himanshu Goel, Richard L. Printz, Chiyo Shiota, Shanea K. Estes, Venkat Pannala, Mohamed Diwan M. AbdulHameed, Masakazu Shiota, Anders Wallqvist

**Affiliations:** 1 Department of Defense Biotechnology High Performance Computing Software Applications Institute, Telemedicine and Advanced Technology Research Center, U.S. Army Medical Research and Development Command, Fort Detrick, Frederick, MD 21702, USA; 2 The Henry M. Jackson Foundation for the Advancement of Military Medicine, Inc., Bethesda, MD 20817, USA; 3 Department of Molecular Physiology and Biophysics, Vanderbilt University School of Medicine, Nashville, TN 37232, USA; 4 Division of Diabetes, Endocrinology and Metabolism, Department of Medicine, Vanderbilt University Medical Center, Nashville, TN 37232, USA

**Keywords:** predictive toxicology, RNA-seq, mercuric chloride, guinea pig, toxicity modules, KEGG pathways

## Abstract

Acute kidney injury, which is associated with high levels of morbidity and mortality, affects a significant number of individuals, and can be triggered by multiple factors, such as medications, exposure to toxic chemicals or other substances, disease, and trauma. Because the kidney is a critical organ, understanding and identifying early cellular or gene-level changes can provide a foundation for designing medical interventions. In our earlier work, we identified gene modules anchored to histopathology phenotypes associated with toxicant-induced liver and kidney injuries. Here, using in vivo and in vitro experiments, we assessed and validated these kidney injury-associated modules by analyzing gene expression data from the kidneys of male Hartley guinea pigs exposed to mercuric chloride. Using plasma creatinine levels and cell-viability assays as measures of the extent of renal dysfunction under in vivo and in vitro conditions, we performed an initial range-finding study to identify the appropriate doses and exposure times associated with mild and severe kidney injuries. We then monitored changes in kidney gene expression at the selected doses and time points post-toxicant exposure to characterize the mechanisms of kidney injury. Our injury module-based analysis revealed a dose-dependent activation of several phenotypic cellular processes associated with dilatation, necrosis, and fibrogenesis that were common across the experimental platforms and indicative of processes that initiate kidney damage. Furthermore, a comparison of activated injury modules between guinea pigs and rats indicated a strong correlation between the modules, highlighting their potential for cross-species translational studies.

## 1. Introduction

Heavy metals, such as arsenic, cadmium, lead, mercury, and uranium, are commonly used for a variety of purposes. However, they are environmental toxicants with the potential to induce acute life-threatening or chronic debilitating health conditions [1,2,3,4]. According to the Agency for Toxic Substance and Disease Registry [5,6], mercury is the third most toxic compound, after arsenic and lead, in terms of its frequency, toxicity, and potential for human exposure. Mercuric compounds are ubiquitous in the environment in different chemical forms, and avoiding exposure can be difficult for both humans and animals. Of the mercury compounds, mercuric chloride involves numerous applications in the preservation of anatomical specimens, photography, leather tanning, and fabric printing and as a chemical reagent and catalyst or disinfectant [7,8,9]. The vapor pressure of mercuric chloride creates an inhalation hazard at normal temperatures, with increasing intensity at higher temperatures. In addition, a small amount of mercuric chloride (0.1 g) can damage body tissues, and doses greater than 1 g can be deadly for an adult human [9]. It is highly soluble in water and can be taken up through inhalation, ingestion, and absorption through the skin. Thus, mercuric chloride is classified as one of the most toxic and corrosive mercury salts and has been associated with serious health issues, affecting the gastrointestinal tract, central nervous system, and renal system [10,11]. Compared to other organs, the kidneys are the prime target of buildup from prolonged exposure to mercuric chloride. The main functions of the kidneys are to filter blood, remove waste products from the body, and maintain the balance of water and solutes. Frequent exposure to mercuric chloride could lead to accumulation in the proximal tubule epithelial cells, which reduces the detoxification and excretion abilities of the kidney, eventually causing acute tubular necrosis and leading to kidney failure [12,13]. Furthermore, mercuric chloride can cause acute renal failure, as described in earlier studies [14,15,16], and is often used as a model toxicant to induce kidney injury [17,18,19,20,21].

Understanding the direct relationship between a toxic chemical and acute kidney or liver injury on the gene level requires a toxicogenomic approach. The field of toxicogenomics involves the aggregation and analysis of gene expression, protein activity, and metabolic profiling (transcriptomics, proteomics, and metabolomics) to reveal molecular patterns of toxicity-induced injuries. Moreover, identifying molecular mechanisms and relevant biomarkers will aid in understanding the toxic liability/safety profile of chemicals and drugs under development. In recent years, our research group has used these approaches to identify significantly expressed genes and potential biomarkers to assess the adverse effects of toxicants and their ability to induce liver and kidney injuries in rats, using in vivo and in vitro experiments [22,23,24,25,26]. Identifying gene-level transcriptional changes induced by a toxic chemical and linking them to a phenotypic process has been accomplished in our previous studies using exemplar toxicants, such as thioacetamide, acetaminophen, bromobenzene, carbon tetrachloride, and mercuric chloride [27,28,29,30,31,32]. In particular, the Web-based gene-set analysis tool TOXPANEL 1.0 [33] assesses eight and 11 injury modules for kidney and liver co-expressed genes, respectively, to identify injury phenotypes derived from the Open Toxicogenomics Project-Genomics Assisted Toxicity Evaluation System (TG-GATES) database [34] or DrugMatrix [35]. Each module is sensitive to the specific phenotype of the organ injury and its severity. TOXPANEL 1.0 modules also support the characterization of the molecular mechanism behind liver and kidney injuries and can be considered as a surrogate biomarker for molecular key events. In summary, these efforts have integrated experimental in vitro or in vivo studies and computational modeling techniques to determine the effects of hazardous chemicals.

In this study, we aimed to evaluate the toxic effects of mercuric chloride on the kidneys of guinea pigs using in vivo and in vitro approaches and to identify similarities in kidney responses between the two platforms. Initially, we performed preliminary dose-response studies and identified the doses (1.00 and 1.50 mg/kg for in vivo and 30 and 40 µM for in vitro) and exposure times (9 and 33 h for in vivo and 8 and 24 h for in vitro) that induce noticeable and consistent alterations in the current clinical chemistry markers for kidney dysfunction or cell viability assays. Then, for the selected doses and time points, we measured the transcriptional changes in the kidney (in vivo) or renal proximal tubular epithelial cells (in vitro) using RNA sequencing and performed toxicogenomic analysis to compare the results at different levels of biological complexity to characterize the mechanisms of kidney injury. In our previous study [31], we reported the time- and dose-dependent effects of mercuric chloride treatment on rats and described how it activated different injury modules that are linked to histopathological kidney changes associated with necrosis, dilatation, degeneration, hyaline casts, and fibrosis. There are a number of studies addressing renal toxicity for different exposures of mercuric chloride in rats [14,15,36,37,38]. Therefore, in the current study, we extended this analysis and explored the interspecies correlation between mercuric chloride injuries induced in guinea pigs and rats through toxicity modules, altered KEGG (Kyoto Encyclopedia of Genes and Genomes) pathways, and gene-level analysis of the transcriptomic response. Moreover, this study also demonstrates the ability of our injury modules to predict kidney injuries in guinea pigs.

## 2. Results and Discussion

### 2.1. Dose Optimization of Mercuric Chloride in Guinea Pigs

**Figure 1:**
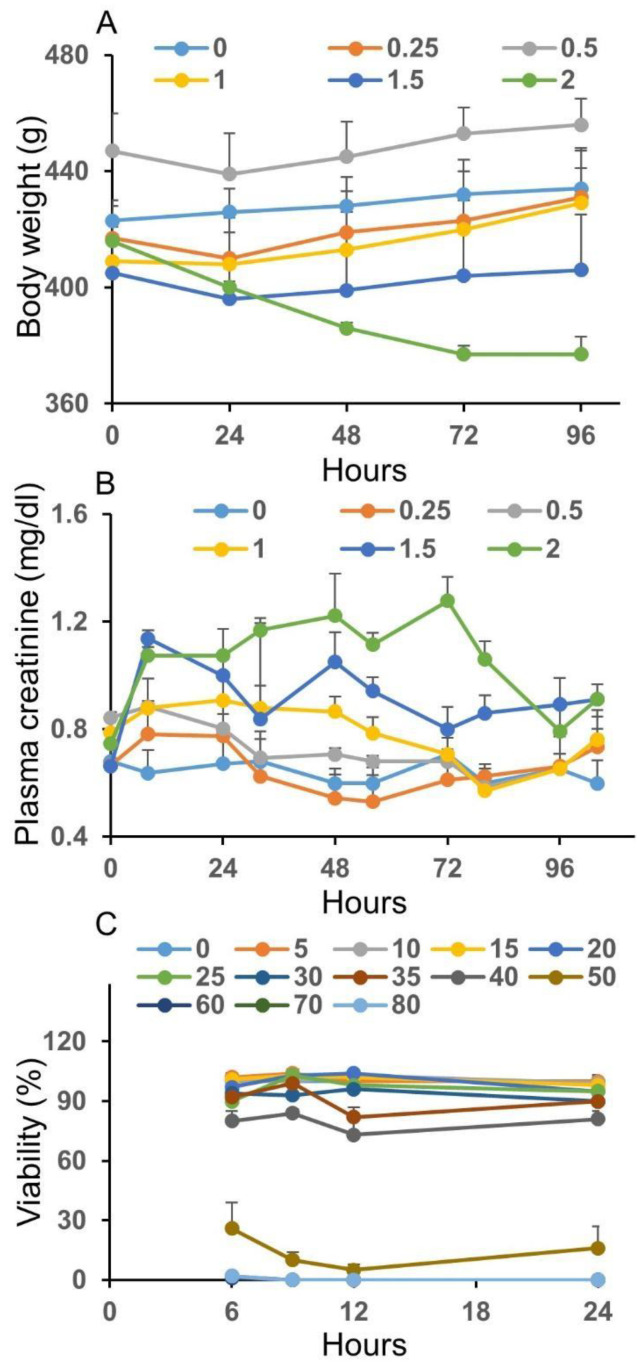
Measurements of injury markers. Effect of a single intraperitoneal injection of mercuric chloride on (**A**) body weight and (**B**) plasma creatinine. (**C**) Effect of mercuric chloride on viability (ATP content) of guinea pig renal proximal tubular epithelial cells. The colored data points and lines represent the responses to the indicated doses of mercuric chloride.

We performed preliminary studies to determine the appropriate low and high doses and times for post-toxicant exposure that can be used to measure the transcriptional changes in the kidney or renal tubular epithelial cells. For the in vivo studies, we exposed guinea pigs to six different concentrations of mercuric chloride, i.e., 0.00 (control), 0.25, 0.50, 1.00, 1.50, and 2.00 mg/kg (n = 3 each), by giving an intraperitoneal injection and monitoring the changes in body weight and plasma creatinine concentrations as shown in Figure 1A,B. Except for the control (0.00 mg/kg) and the highest dose of mercuric chloride (2.00 mg/kg), the body weight started to decrease nominally during the initial 24 h and increased later. For the control group, the body weight steadily increased, and for the highest dose, it decreased continuously until 72 h, signifying a more significant physical injury. Similarly, the plasma creatinine level for the control group did not show marked variation, whereas it steadily increased until 72 h for the highest dose. Such a rapid increase in the plasma creatinine concentration is known to be a characteristic feature of the acute kidney injury (AKI) [39,40]. Mercuric chloride doses of 0.25, 0.50, and 1.00 mg/kg do not appear to have caused injuries to the kidneys, as reflected by the absence of a significant body weight loss and relatively small changes in plasma creatinine. We did not observe any significant changes in plasma creatinine levels between 0 and 32 h after doses of 0.25 and 0.50 mg/kg. The plasma creatinine levels increased within 8 h for most of the doses but eventually reached normal levels of variation. However, plasma creatinine levels increased significantly with the 1.50 and 2.00 mg/kg doses, indicating potential complications, whereas the 1.00 mg/kg dose induced moderate changes. Therefore, we selected a low dose to represent the highest dose that could lead to mild or no organ injury and a high dose to represent the lowest dose that could result in organ injury as reflected by plasma creatinine alterations. While considering all these factors, we selected a single intraperitoneal injection of 1.00 and 1.50 mg/kg as the appropriate low and high doses of mercuric chloride and 9 and 33 h as the two timepoints.

For the in vitro studies, we examined a total of 13 doses of mercuric chloride (n = 6 each), i.e., 0 (control), 5, 10, 15, 20, 25, 30, 35, 40, 50, 60, 70, and 80 µM, with regard to cell viability (ATP content) of guinea pig renal proximal tubular epithelial cells. Figure 1C shows the responses to all of the doses. Similar to the in vivo study, we selected a low dose to represent the highest concentration leading to mild or no cell damage and a high dose to represent the lowest concentration resulting in apparent cell death. Our criterion for the high dose was to achieve a ≤20–30% drop in cell viability at the longer exposure times with respect to control. The 5, 10, 15, 20, and 25 µM doses did not induce significant variations in cell viability as compared to the response to the control dose. On the other hand, the 50, 60, 70, and 80 µM doses caused marked cell death and were not pursued further. The 30, 35, and 40 µM doses led to an ~5–20% decrease in cell viability. Because the cell viability response was severe for the 40 µM dose, we selected 30 µM as the low dose and 40 µM as the high dose and exposure times of 12 and 24 h.

### 2.2. Gene-Level Analysis

The impact of toxicant exposures can be quantified by the number of genes significantly altered within a certain time duration in each experiment. Table 1 summarizes the number of significantly altered differential expression genes (DEGs) and their overlap count from the in vivo and in vitro experiments alone and their similarities across the conditions. The criterion to identify DEGs was based on a q-value of <0.1. The relatively high number of DEGs identified for the in vivo and in vitro studies highlights the deleterious impact of mercuric chloride on the kidneys. As the dose of the toxicant increased (from 1.00 to 1.50 mg/kg for in vivo and from 30 to 40 µM for in vitro), the number of DEGs increased from 4272 to 5665 for the in vivo and from 4377 to 7540 for the in vitro experiments at the shorter time intervals of 9 h and 12 h, respectively. However, as the time increased, the effect of the toxicant at the low dose was lessened, as reflected by the reduced number of DEGs compared with the shorter time intervals. In contrast, in the in vivo studies with the high dose of mercuric chloride, the DEGs count increased from 5665 to 8264, signifying an increased effect on the kidneys with time. Moreover, we observed a similar trend in our previous study of thioacetamide-induced kidney injury [27]. These results indicate that at low concentrations, the guinea pigs were able to cope with the mercuric chloride toxicity and recovered as time progressed, but they may have incurred severe damage at higher concentrations as reflected by the larger changes in the DEGs with higher doses, indicating kidney injury. These results show the utility of significant DEGs in capturing molecular-level changes in toxic injuries. In addition, we observed a large number of overlapping genes within the in vivo or in vitro experiments for different dose and time conditions (Table 1). We observed that the highest number of significant DEGs overlapped (4679) for the high dose at the longer duration in vivo and with the shorter duration in vitro between the two platforms. We also observed considerable overlap of DEGs at the high dose between both platforms at the longer duration. The overall overlap of genes between both sets of experiments indicates a possible commonality of kidney injury and linkage of genes to injury phenotypes.

To ascertain the impact of these overlapped DEGs between the two platforms on kidney function, we performed a KEGG pathway analysis of the 2825 overlapping genes between the in vitro and in vivo experiments (at the high dose and longer time point) using the DAVID Gene Functional Classification Tool [41]. Table 2 shows the 15 most enriched KEGG pathways that are common in both sets of experiments, with the cell cycle pathway at the top of the list. Many of these pathways are previously known to be involved in AKI. For example, when tubular epithelial cells come in contact with toxic chemicals, cell death normally ensues, with closely related necrotic and apoptotic processes associated with AKI [42,43,44].

The extracellular matrix-receptor interaction, focal adhesion pathways, and regulation of the actin cytoskeleton are well-known disease processes associated with AKI [45,46,47]. The p53 pathway plays a pathological role in cisplatin-induced AKI [48,49]. In addition, glutathione metabolism has a key role in oxidative stress events, contributing to tubular cell injury [50,51]. Furthermore, several of the other pathways listed in Table 1, such as DNA replication, metabolic, phosphatidylinositol 3-kinase (PI3K)-Akt signaling, and tumor necrosis factor (TNF) signaling, are also reported to be associated with renal diseases [52,53,54,55,56,57].

**Figure 2:**
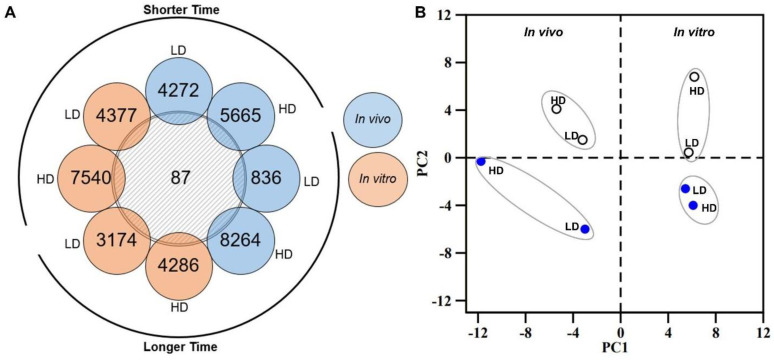
(**A**) Venn diagram depicting common differential expression genes (DEGs) between different times of exposure and doses of mercuric chloride for in vivo and in vitro studies. (**B**) Principal component analysis (PCA) for 87 common DEGs obtained from all conditions in the in vivo and in vitro data. The in vivo and in vitro conditions are presented on the left and right panels, respectively. The white and blue circles represent shorter and longer times of exposure, respectively. HD, high dose; LD, low dose.

To further characterize the relevance of significantly altered DEGs and their potential role in kidney function and disease, we compared DEGs across all conditions and identified the list of genes that are common across the platforms and conditions. Figure 2A shows the count of the 87 common genes among each DEG set observed in both in vivo and in vitro experiments for all combinations of low/high doses and shorter/longer times of exposure. The presence of these overlapping genes in the mercuric chloride exposure datasets allows us to examine a possible common correlation among the examined conditions. For this purpose, we performed a principal component analysis (PCA) with these 87 genes and the corresponding fold-change values from all the in vitro and in vivo dose/time datasets. The PCA plot in Figure 2B shows four different clusters in different quadrants, clearly separating all in vivo and in vitro experiments as well as shorter and longer exposure times. This analysis did not allow us to identify any commonality or strong correlation among the conditions.

By examining the individual averaged and sorted fold-change values from all eight sets of 87 genes, we assessed individual gene up- or downregulation to better understand the mapping of genes to kidney injuries. Table 3 shows the top 10 up- and downregulated genes, indicating a common involvement of these genes in mercuric chloride exposure in guinea pigs. For example, the upregulation of the glutathione-*S*-transferase (Gsta2, Gsta5, Gsto1, and Gstp1) family of genes was found in the erythrocytes of patients with chronic renal failure [58]. These genes provide protection for cells against reactive oxygen species, and increased expression has been correlated with the cytotoxicity [59,60,61]. The metallothionein genes (Mt2A, Mt1m) have also been associated with renal tissues and play a protective role [62,63,64]. The additional upregulated genes listed in Table 3, such as Uchl1, Rrm2, and Aldh1a7, are also closely connected with AKI pathways [60]. On the other hand, the top downregulated gene among the experimental set was slc7a13, which has been identified in AKI conditions [65,66,67]. Other downregulated gene sets (cbr1, ppp1r3c, smoc1, cd200, and Col11a1) have also been connected with acute or chronic kidney injury [60,68,69]. In summary, exploring genetic information and highlighting the common genes among different experimental models offers a reasonable approach to characterize kidney injury mechanisms.

### 2.3. Kidney Injury Module Activation Analysis

**Figure 3:**
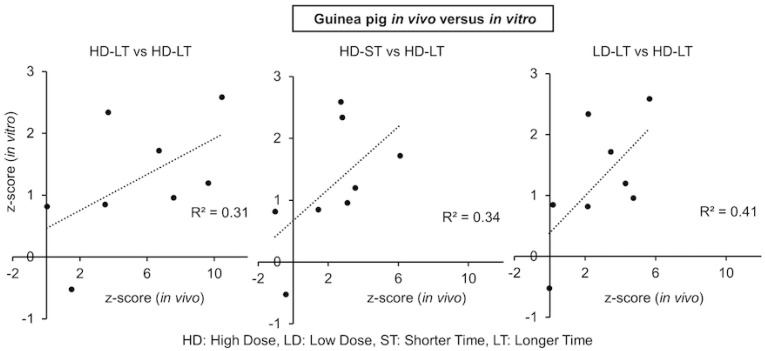
Correlation between kidney injury module scores for the in vivo versus in vitro guinea pig studies.

In our earlier work, we formulated eight kidney injury and 11 liver injury modules, each consisting of multiple sets of genes associated with the histopathological kidney or liver injury phenotypes, and all the functionality of these modules and gene analyses are accessible through the TOXPANEL software 1.0 [33]. We used TOXPANEL 1.0 with the guinea pig genomic data to analyze the responses of the co-expressed genes and assess the severity of kidney injury. Table 4 lists the activation scores (z-scores) for all eight kidney injury modules in guinea pigs for both sets of experiments (in vivo and in vitro) at two doses of mercuric chloride and two assessment times. The degree of activation of the different modules was assessed via the z-score metric, and values >2 were regarded as a significant indication of injury. The in vivo experiment results essentially showed activation of the five modules, specifically *Dilatation, Hyaline Cast, Degeneration, Necrosis,* and *Fibrogenesis,* for the low and high doses at the shorter or longer exposure times. As the dose increased, most of the module scores showed higher z-scores, signifying a strong dependence on the dosage. Similarly, many of the modules’ z-scores increased as time progressed for the in vivo study, which is in qualitative agreement with earlier observations [27,28,29,31]. In general, the modules from the in vivo study showed stronger activation at higher doses and longer time points, but we noted the activation of these modules at the lower dose and shorter time points as well. However, the trend was the opposite with the z-scores for the in vitro outcomes. The z-scores for most of the modules in the in vitro study displayed much lower activation across different dose and time conditions as compared with the in vivo z-scores. For the in vitro experiments, we observed the activation of the *Dilatation, Necrosis, Cellular Infiltration, Fibrogenesis,* and *Cellular Infiltration* modules for the high dose of mercuric chloride at the shorter exposure time. However, most of the module activation declined as time increased, except for *Dilatation* and *Fibrogenesis.* The activation of some of the modules occurred at a lower dose and shorter time; however, all module scores abated by 24 h. To further understand the correlation between both sets of experiments, Figure 3 shows three correlation plots of module scores for different doses and times of exposure. Because it is difficult to compare and obtain correlation metrics for different experimental systems, doses, and exposure times, it is encouraging that we observed a fair correlation (*R^2^*~0.31) between the high dose and longer time (HD-LT) conditions for both in vitro and in vivo experiments. Furthermore, different time and dose conditions also demonstrated a fair correlation between the module scores of the experimental systems.

### 2.4. Correlation between Guinea Pig and Rat Mercuric Chloride Exposures

Our earlier work [31] examining rat in vivo exposure to mercuric chloride showed the activation of the *Dilatation, Hyaline Cast, Degeneration, Necrosis,* and *Fibrogenesis* modules at the higher dose and longer exposure time (34 h). Hence, it is of interest to examine the inter-species correlation between guinea pig and rat mercuric chloride exposure. The guinea pig in vivo results in this study also demonstrated the activation of all these modules to a similar or greater degree at the higher dose and longer time (33 h). The similarity of the module activation between the two studies indicates a similar type of molecular response mechanism or pathway behind the injury phenotype activity. In the rat in vivo study, the *Necrosis* and *Dilatation* modules were also activated with less intensity at the higher dose and shorter exposure time (10 h). In addition, the activation of the modules was not seen for the low dose of mercuric chloride at 10 h or 34 h after treatment. However, in this guinea pig in vivo study, we did observe the activation of most of these five modules for a shorter time of exposure at low or high doses. Comparably, the *Dilatation, Necrosis,* and *Fibrogenesis* modules also showed activation in the in vitro guinea pig study at the high dose and shorter exposure time (12 h). As time progressed, cells started to decay, and only the *Dilatation* and *Fibrogenesis* modules remained activated at the 24-h exposure time. Previous studies of rodent mercuric chloride exposure have shown that the largest impact can be seen on the oxidative stress, morphological alterations, and inflammatory responses, which involve the hallmark degeneration proceedings related to *Necrosis, Dilatation, Fibrosis, Degeneration,* and *Hyaline Casts* modules, in accordance with the known histopathology associated with mercuric chloride [13,14,17,18,20,21,36,38,55,70].

**Figure 4:**
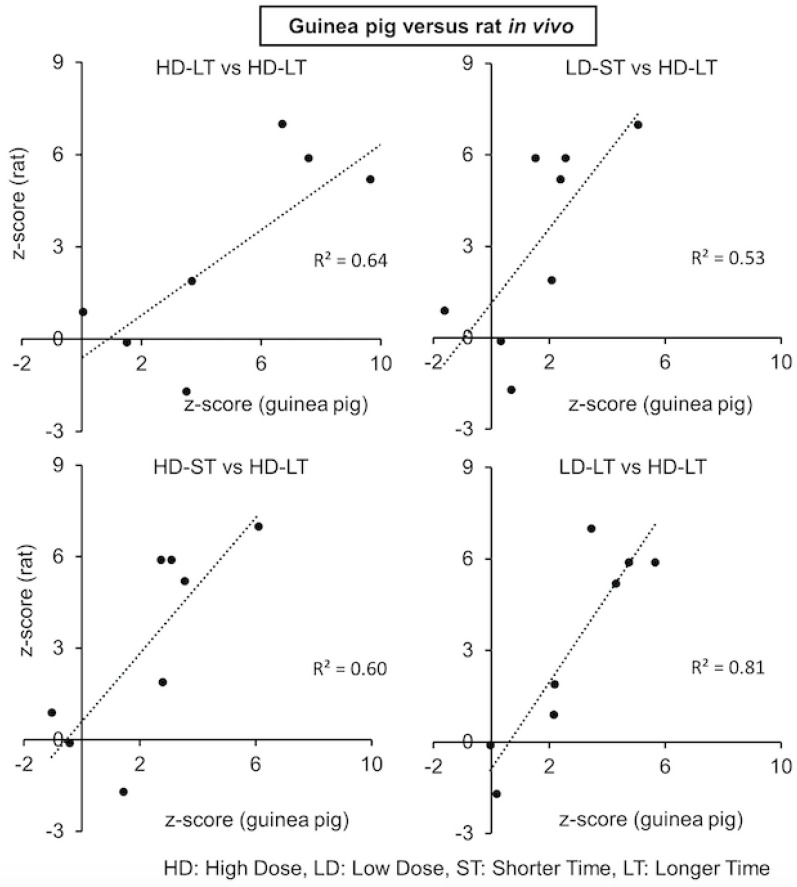
Correlation between the kidney injury modules for guinea pig versus rat in vivo data.

Figure 4 shows the correlation plots of module z-scores for different doses and exposure times for the guinea pig and rat studies. We observed a correlation score of *R^2^*~0.64 for the high dose and longer exposure time between the rat and guinea pig in vivo data. Similarly, different exposure times and dose conditions also demonstrated strong correlations of the module scores (*R^2^*~0.81 for low dose-longer time vs. high dose-longer time) between both sets of experiments. Furthermore, we also used the module score of the rat in vivo and guinea pig in vivo and in vitro data to identify the correlation between the experimental outcomes for doses and exposure times.

**Figure 5:**
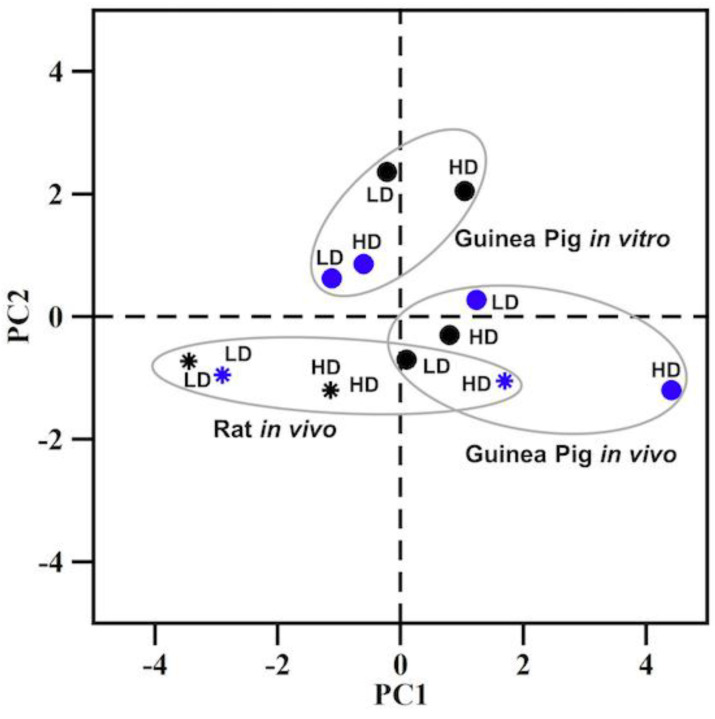
Principal component analysis (PCA) for kidney injury module activation for the different conditions of guinea pig (in vivo and in vitro) and rat data (in vivo) [31]. The circles and asterisks represent samples from guinea pigs and rats, respectively. The black and blue symbols represent the shorter and longer exposure times, respectively. HD, high dose; LD, low dose.

Figure 5 shows the PCA plot of the three clusters representing the three different experimental groups. The PCA plot indicates a clear division of clusters between the in vitro data and the rat/guinea pig in vivo data. There is an overlap of the cluster signifying robust correlations between the rat and guinea pig in vivo data. The data for the rat in vivo higher dose and longer exposure time are in close proximity to the guinea pig in vivo data. Furthermore, for the rat or guinea pig in vivo datasets, the data for the high dose with the longer exposure time are positioned at the extreme right of the respective clusters as compared with all the data within the cluster, indicating heightened injuries in both animal models. These findings demonstrated a relational capability and injury sensitivity of using modules to assess and understand the interspecies correlation with respect to experimental model, dose, and time considerations.

### 2.5. KEGG Pathway Analysis

**Figure 6:**
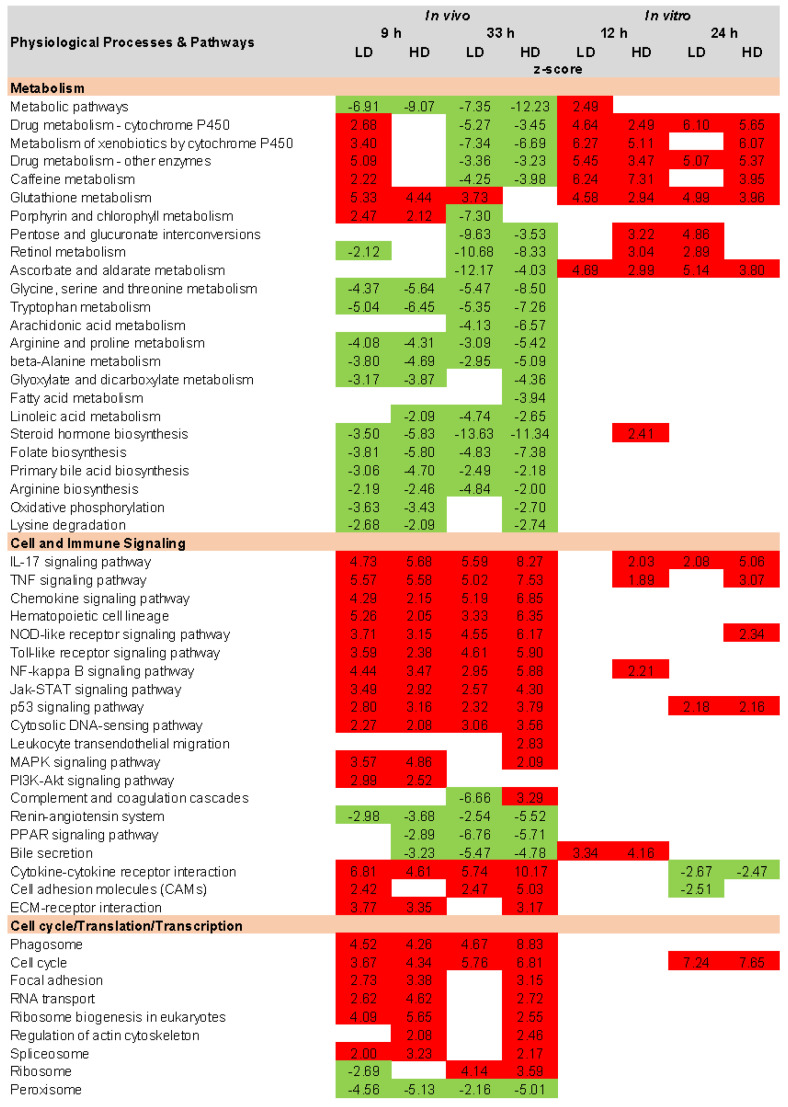
KEGG (Kyoto Encyclopedia of Genes and Genomes) pathways activated using gene expression data following guinea pig mercuric chloride exposure. Significantly up- and downregulated pathways are indicated by red and green, respectively. HD, high dose; LD, low dose.

The development of AKI involves multiple cellular processes, signaling pathways, inflammatory responses, and molecular mechanisms. Identification and interpretation of the physiological processes and pathways associated with toxicant-induced kidney injury aid in delineating known and unknown AKI mechanisms. The TOXPANEL software 1.0 [33] uses the AFC method [71,72] to calculate and display the KEGG upregulated and downregulated pathways. Figure 6 shows all the key KEGG pathways identified in the guinea pig in vivo and in vitro experiments for all the conditions examined here. We categorized the pathways into three different groups, i.e., *Metabolism, Cell and Immune Signaling*, and *Cell Cycle/Translation/Transcription*. The significantly upregulated and downregulated pathways are shown by their z-scores and are color coded for all the dose and exposure time conditions. Figure 6 indicates that most of the metabolic pathways in the in vivo study were downregulated for carbohydrate, amino acid, global, lipid, and xenobiotic metabolism at shorter and longer exposure times for both doses of mercuric chloride. However, these trends were different for the in vitro data, which showed primarily upregulated pathways. While information on AKI and its association with metabolic pathways is limited in the available literature, the cytochrome P450 family of hemoproteins has been shown to be closely involved in the oxidative metabolism of drugs [73]. In addition, different clinical studies showed the impairment of cytochrome and transporter activity in kidney injury processes [74,75] and that an adequate supply of glutathione is crucial for maintaining kidney function and helps in detoxifying the kidneys [76,77]. Our results showed upregulation for some pathways, i.e., *Drug Metabolism, Cytochrome P450, Metabolism of Xenobiotics, Other Enzymes*, and *Glutathione Metabolism,* at a shorter time period. However, as time progressed, most of the metabolism pathways became downregulated. The downregulated trend of the metabolism-related pathways was consistent with the experimental findings, indicating impaired renal function [56,78,79,80].

The inflammatory response is critical in AKI pathogenesis and is regularly associated with increased cytokines, lung chemokine upregulation, and neutrophilic infiltration [81]. The cytokines play an essential role in the activation or differentiation of inflammatory and immune responses and also have pro-inflammatory and anti-inflammatory properties [82]. Cytokines and chemokines are key pathways for regulating inflammation, immune responses, and cellular signaling. Our results for most of the cell and immune signaling pathways displayed upregulation for guinea pigs in vivo and in vitro, consistent with our earlier rat mercuric chloride study [31]. A number of potent mediator pathways for epithelial proximal tubular cell injury and AKI involves TNF, Toll-like receptor (TLR), cytokine-cytokine receptor interaction, interleukin-17, p53, PI3K-Akt, mitogen-activated protein kinase, focal adhesion, cell cycle, regulation of actin cytoskeleton, cell adhesion molecules, complement and coagulation cascade, and chemokine signaling [23,44,45,46,49,55,56,57,70,83,84,85,86,87,88,89]. All these signaling pathways are involved in the cellular processes of proliferation, apoptosis, and adhesion. The upregulation of these pathways, especially at the later time point, is in overall agreement with the known AKI pathophysiology and indicative of kidney injury.

## 3. Materials and Methods

### 3.1. Experimental Design for In Vivo Studies

#### 3.1.1. Animals

We purchased 4-week-old male Hartley guinea pigs from Charles River Laboratories (Wilmington, MA, USA). We fed them a normal diet (No. 5025, LabDiet, St. Louis, MO, USA) and provided water ad libitum in an environmentally controlled room with a 12:12-h light-dark cycle and the temperature set at 23 °C. All experiments were conducted in accordance with the Guide for the Care and Use of Laboratory Animals of the United States Department of Agriculture, the Vanderbilt University Institutional Animal Care and Use Committee, and the U.S. Army Medical Research and Development Command Animal Care and Use Review Office.

Guinea pigs were kept for 7 days prior to any treatment to recover from the stress of conveyance and to acclimate to their new housing environment. Then, we anesthetized them with isoflurane and implanted a sterile silicone catheter (0.5 mm inner diameter, 0.94 mm outer diameter) into the right jugular vein. The free end of the catheter was passed subcutaneously to the back of the neck, where it was fixed and occluded with a metal plug following a flush of heparinized saline (200 U heparin/mL saline) [90]. After the procedure, animals were housed individually in a metabolic cage (Harvard Apparatus, Holliston, MA, USA) for 7 days to recover from the stress associated with the surgery before we began the studies for optimization of mercuric chloride dose and length of exposure and for measuring the toxicant-induced changes in gene expression.

#### 3.1.2. Preliminary Studies for Optimization of Dose and Time after Exposure

To determine the appropriate dose of mercuric chloride and time after exposure, we divided the animals into six groups (n = 3 per group) and treated them with either vehicle (1 mL/kg saline) or mercuric chloride (0.25, 0.50, 1.00, 1.50, or 2.00 mg/kg, injected intraperitoneally at 9 a.m.). We collected blood samples (150 µL) from the jugular vein catheter under unanesthetized and unrestricted conditions just before as well as 8, 24, 32, 48, 56, 72, 80, 96, and 104 h after the dosing treatment. After the last blood collection, we euthanized the guinea pigs by intravenous administration of sodium pentobarbital (120 mg/kg) through the jugular vein and harvested the kidneys. We quantified plasma creatinine as a measure of kidney functionality using a creatinine assay kit (ab65340, Abcam Inc., Waltham, MA, USA).

#### 3.1.3. Studies for Measuring Changes in Gene Expression

**Figure 7:**
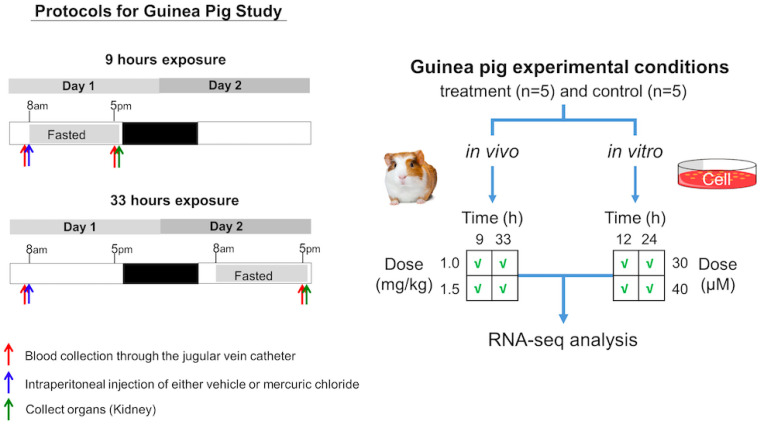
Summary of the experimental conditions used in the guinea pig mercuric chloride exposure studies for either in vivo animal or in vitro cell-based studies.

Based on the results of preliminary studies to optimize the dose and time after exposure, we selected 1.00 and 1.50 mg/kg as the low and high doses, respectively, and 9 and 33 h as the time elapsed after mercuric chloride exposure (Figure 7). In mammals, the expression and/or activity of key metabolic enzymes, transcription factors, signaling molecules, and transporters display circadian rhythmicity, with an oscillation of 24 h, which is driven by the light-dark cycle and controlled by a central nervous system (CNS) and peripheral circadian clock system. The feeding cycle controlled by the CNS clock is dominant over the clock associated with the liver, thus affecting the circadian oscillation of lipid, glucose, bile, and drug metabolism in the liver. The circadian oscillation affects gene expression and metabolism in the kidney [91]. In addition, absorption of nutrients that may occur during a small feeding event during the fasting period of a feeding cycle would affect metabolite and hormonal concentrations in the blood as well as renal metabolism. Therefore, to avoid the influence of circadian oscillation and irregular feeding on our measurements, we set the collection time of blood and kidneys at 5 p.m. and fasted animals for 8 h, from 9 a.m. to 5 p.m., in the light phase of the light-dark cycle prior to sampling.

As shown in Figure 7, on Day 1, for both the 9- and 33-h exposure times, following blood collection, we treated the animals with either vehicle or mercuric chloride via intraperitoneal injection at 8 a.m. Then, in the 9-h exposure study, we allowed animals access to water ad libitum but not to food after 8 a.m. In the 33-h exposure study, animals were allowed access to water and food ad libitum for the first 24 h after treatment, and then food was removed at 8 a.m. on Day 2. At 5 p.m. on Day 1 of the 9-h exposure study and on Day 2 of the 33-h exposure study, following blood collection through the jugular vein catheter, we anesthetized the animals by intravenous injection of sodium pentobarbital (50 mg/kg) through the jugular vein catheter and immediately subjected them to a laparotomy. We dissected the kidneys and froze them using Wollenberger tongs precooled in liquid nitrogen. The collected plasma and kidneys were kept in a −80 °C freezer until analysis. Frozen whole kidneys were powdered in liquid nitrogen since these organs are histologically heterogeneous. Total RNA was isolated from the powdered kidneys using TRIzol Reagent (Thermo Fisher Scientific, Waltham, MA, USA) and the direct-zol RNA MiniPrep kit (Zymo Research, Irvine, CA, USA).

### 3.2. Experimental Design for In Vitro Studies

#### 3.2.1. Animals

We purchased 4-week-old male Hartley guinea pigs from Charles River Laboratories (Wilmington, MA, USA). We fed them Guinea Pig Chow 5025 (LabDiet, St. Louis, MO, USA) and provided water ad libitum in an environmentally controlled room with a 12:12-h light-dark cycle at room temperature. All experiments were conducted in accordance with the Guide for the Care and Use of Laboratory Animals of the United States Department of Agriculture, the Vanderbilt University Institutional Animal Care and Use Committee, and the U.S. Army Medical Research and Development Command Animal Care and Use Review Office.

#### 3.2.2. Guinea Pig Renal Proximal Tubular Epithelial Cell Isolation and Culture

We isolated the renal proximal tubules from 6- to 8-week-old guinea pigs using a substantially modified version of the method previously reported for the isolation of proximal tubular epithelial cells from rabbits [92]. We anesthetized the guinea pigs by intraperitoneal injection of sodium pentobarbital (50 mg/kg) and performed a midline abdominal incision. Following ligations of the superior mesenteric artery and the aorta directly below the celiac artery, we immediately cannulated the aorta at the distal site of the left renal artery. We perfused the kidneys in a flow-through manner with ice-cold Dulbecco’s modified Eagle’s medium (DMEM)/F12 at a flow rate of 28 mL/min for 5 min, followed by perfusion with an ice-cold iron oxide particle solution (0.75 g iron oxide particles/15 mL DMEM/F12) at a flow rate of 1 mL/s for 15 s. We drained the effluent through the vena cava during these perfusions and harvested and decapsulated the kidneys following the perfusions. We separated the entire renal cortex from the medulla and cut it into approximately 1-mm^2^ pieces in ice-cold DMEM/F12 solution. We smashed the cubes on a 380-µm metal sieve using a glass bar, and the material passing through the mesh was maintained as a suspension in 100 mL ice-cold DMEM/F12 medium. We removed glomeruli containing iron oxide from the suspension using a magnetic stir bar. Following filtration of the resulting suspension through a 40-µm nylon mesh, we resuspended the purified proximal tubules retained by the mesh in 20 mL of fresh DMEM/F12 medium containing 0.0025% soybean trypsin inhibitor (Type 1-S, Sigma-Aldrich, St. Louis, MO, USA) and 1 mg collagenase H (Worthington, Lakewood, NJ, USA) and incubated them for 3 min at 37 °C. We refiltered the suspension through a 40-µm nylon mesh and resuspended the proximal tubes retained by the mesh in 20 mL of epithelial cell medium (Epi CM-A, ScienCell Research Laboratories, Carlsbad, CA, USA). We plated the suspended proximal tubes onto two 10-cm poly-D-lysine-coated plates and maintained them at 37 °C in a humidified 5% CO_2_ incubator for 10–12 days to allow tubular epithelial cells to form a tight “cobblestone-like” monolayer. The epithelial cells were harvested using 0.05% trypsin-EDTA solution (25300, Thermo Fisher Scientific), collected with Epi CM-A, and centrifuged at 600× *g* for 10 min. We resuspended the resulting cell pellet of epithelial cells in Epi CM-A and plated the cells onto poly-D-lysine-coated 96-well plates at 2 × 10^4^ cells/well for measurement of cell viability and onto poly-D-lysine-coated 6-well plates at 3 × 10^5^ cells/well for RNA isolation. After 48 h of culture to allow cell reattachment, we changed the cell culture medium to the Hepatocyte Maintenance Medium (CC-3198, Lonza, Basel, Switzerland) for 48 h before exposure to mercuric chloride.

#### 3.2.3. Preliminary Studies to Ascertain the Optimal Exposure of Toxicant

To optimize the dose and length of exposure to mercuric chloride, we exposed guinea pig renal proximal tubular epithelial cells to vehicle or multiple concentrations of mercuric chloride ranging from 0 to 80 µM (n = 6 for each) for various lengths of time (6, 9, 12, and 24 h). After the cells were exposed to vehicle or mercuric chloride, we assessed cell viability by measuring cellular ATP content for each dose and time combination using the CellTier-Glo 2.0 assay kit (Promega, Madison, WI, USA) according to the protocol of the manufacturer.

#### 3.2.4. Exposure of Cells to Toxicant for RNA Isolation

Based on the results of preliminary studies, we chose 30 and 40 µM as the low and high doses of mercuric chloride for renal proximal tubular epithelial cell exposure. We also selected 12 and 24 h as the shorter and longer lengths of exposure to mercuric chloride (Figure 7). To extract RNA from the cultured cells for each combination of dose and time, we removed the culture medium, added 1 mL TRIzol (Thermo Fisher Scientific, Waltham, MA, USA) to each well of cells, and harvested the lysed cells in the TRIzol solution. We further purified the RNA collected from the aqueous phase of TRIzol using the RNeasy Plus Mini kit (Qiagen, Germantown, MD, USA) and eliminated genomic DNA by in-column DNase I digestion.

### 3.3. RNA Sequencing

We submitted the isolated RNA samples from the in vivo and in vitro studies to the Vanderbilt University Medical Center VANTAGE Core (Nashville, TN, USA) for RNA quality determination and sequencing. Total RNA quality was assessed using a 2100 Bioanalyzer (Agilent, Santa Clara, CA, USA). At least 200 ng of DNase-treated total RNA with high RNA integrity were used to generate poly-A-enriched mRNA libraries using KAPA-stranded mRNA sample kits with indexed adaptors (Roche, Indianapolis, IN, USA). Library quality was assessed using the 2100 Bioanalyzer (Agilent), and libraries were quantitated using KAPA library quantification kits (Roche). Pooled libraries were subjected to 75-bp paired-end sequencing for in vivo studies and 150-bp pair-end sequencing for in vitro studies according to the manufacturer’s protocol (Illumina NovaSeq6000, San Diego, CA, USA). Bcl2fastq2 Conversion Software (Illumina) was used to generate demultiplexed Fastq files. All the files obtained from the RNA-seq are deposited in the NCBI’s Gene Expression Omnibus (GEO) database, using GEO series accession number GSE226313.

### 3.4. Analysis of RNA-seq Data

To analyze the RNA-seq data, we used the Kallisto tool [93], which involves the pseudo-alignment of reads to a reference genome and quantification of the transcript abundances for each read. Here, we pseudo-aligned the reads to the *Cavia porcellus* transcriptome downloaded from the Ensemble website [94]. The Kallisto analysis is comparable to other challenging methods in terms of accuracy and computational time expense [95]. The bootstrapping technique involving estimation for the uncertainty of transcript abundance was handled by repeating the analyses 100 times after resampling with replacement. In addition, to identify the DEGs, we used Kallisto’s companion analysis tool Sleuth [96], which uses the transcript abundance data obtained from Kallisto to estimate the technical gene variance for each sample. We also set the criteria for the significantly expressed genes with a false discovery rate-adjusted *p*-value (q-value) of ≤0.1. All the genes and their associated q-values and fold changes can be found in the Supplementary Materials.

### 3.5. Injury Module Activation and Pathway Analysis

We employed the publicly available gene expression-based injury module analysis web tool TOXPANEL 1.0 to assess kidney injury activation from the guinea pig in vitro or in vivo genomic data. This tool uses the aggregated absolute fold-change (AAFC) and aggregate fold-change (AFC) methods developed previously by our research group [27,28]. The genomic data consist of a set of genes for control and treatment cohorts and their log-transformed gene-expression values. The AAFC method calculates the fold changes by taking the difference between the mean log-transformed gene-expression values for all the samples in the treatment and control cohorts. This results in the identification of a set of genes that are significantly changed, with a fold-change value for each gene, and is subsequently reflected in the z-score of each module or pathway encompassing several gene sets with their fold-change values. The AAFC method only accounts for the change in the set of genes and does not identify up- or downregulated genes. The AFC method can provide the directionality of gene expression (sum of the up- or down-expressed genes), which becomes useful for KEGG pathway analysis [97]. Readers are referred to earlier articles [71,72] for additional details about the AFC methodology.

We estimated the significance of each module and set of genes using its *p*-value, which is essentially defined as the probability that the score for randomly selected fold-change values (repeated 10,000 times) is greater than the score from the actual module [33]. A *p*-value < 0.05 signifies the importance of the module. The z-score parameter, which is the number of standard deviations by which the actual module value changes from the mean of the arbitrarily selected fold-change values (repeated 10,000 times), specifies the gradation of module activation and signifies the role of different modules and their severity when subjected to a toxicant [33].

## 4. Conclusions

We examined mercuric chloride-induced kidney injuries in guinea pigs using a combination of in vivo and in vitro experimental and computational modeling techniques, compared the guinea pig studies with similarly developed rat studies, and explored the underlying toxicogenomic signals for the dose-dependent injuries. We analyzed RNA-seq data from mild and severe kidney injuries to identify common genes among the studies, enrichment of AKI-relevant KEGG pathways, and activation of gene modules as a means to gauge interspecies toxicant–response correlations. A large number of DEGs in the in vivo and in vitro experiments were directly related to previously annotated kidney injury-related genes, with the overall overlap among them highlighting the inherent similarity among genes and pathways that were impacted by the model kidney toxicant. The developed kidney-injury gene modules in the TOXPANEL 1.0 application comprise sets of co-expressed genes correlated with specific injury phenotypes, capturing genes directly or indirectly associated with molecular toxicity and pathways relating to the histological damage of the kidney. Specifically, we observed the activation of the *Dilatation, Hyaline Cast, Degeneration, Necrosis,* and *Fibrogenesis* modules in the in vivo guinea pig study at different dose and exposure time conditions, consistent with our earlier studies of rat mercuric chloride exposure and with known mechanisms of AKI. The modules displayed a dose-dependent response with significantly higher activation at the higher as compared to the lower dose. The sensitivity of injury module activation based on gene expression changes was evident in both the in vitro and in vivo experimental platforms and provided an indication of kidney damage even at the low dose and short exposure times, indicating that injury modules could also serve as a tool for predicting kidney injuries. In addition, the KEGG pathway enrichment analysis revealed the activation of several pathways and inflammatory processes associated with the activation of kidney injury modules. Altogether, the TOXPANEL 1.0 modules offer the benefit of predicting histopathological damage while offering the advantage of quantitively characterizing the underlying mechanisms of cellular damage. In the end, we plan to directly relate our findings to humans by expanding our interspecies comparisons to include in vitro studies of human cells to identify gene expression data, injury modules, and pathways that are similar.

## Figures and Tables

**Table 1:** Overlap of the number of significantly altered differential expression genes (q < 0.1) found in guinea pigs in vivo and in vitro data across all dose and time conditions. HD, high dose; LD, low dose.

Common Differential Expression Genes (DEGs)						
In vivo	In vivo					
	Overlap		9 h		33 h	
			LD	HD	LD	HD
	9 h	LD	4272	3814	452	3296
		HD		5665	542	4211
	33 h	LD			836	784
		HD				8264
In vitro	In vitro					
	Overlap		12 h		24 h	
			LD	HD	LD	HD
	12 h	LD	4377	3698	2240	2346
		HD		7540	2588	3246
	24 h	LD			3174	2397
		HD				4286
In vivo	In vitro					
	Overlap		12 h		24 h	
			LD	HD	LD	HD
	9 h	LD	1808	2650	1275	1534
		HD	2250	3441	1676	2041
	33 h	LD	332	466	248	289
		HD	2877	4679	2171	2825

**Table 2:** KEGG (Kyoto Encyclopedia of Genes and Genomes) pathways enriched in overlapping differential expression genes.

KEGG Pathway	Benjamini *p*-Value
Cell cycle	3.8 × 10^−11^
ECM-receptor interaction	1.2 × 10^−8^
DNA replication	7.6 × 10^−8^
Metabolic pathways	1.8 × 10^−6^
Focal adhesion	2.2 × 10^−6^
PI3K-Akt signaling pathway	2.4 × 10^−6^
Fluid shear stress and atherosclerosis	2.4 × 10^−6^
p53 signaling pathway	5.7 × 10^−5^
Glutathione metabolism	8.9 × 10^−5^
Regulation of actin cytoskeleton	3.9 × 10^−3^
Drug metabolism—other enzymes	8.2 × 10^−3^
TNF signaling pathway	8.2 × 10^−3^
Glycine, serine, and threonine metabolism	1.6 × 10^−2^
Peroxisome	1.7 × 10^−2^
Chemical carcinogenesis—reactive oxygen species	3.2 × 10^−2^

ECM, extracellular matrix; PI3K, phosphatidylinositol 3-kinase; TNF, tumor necrosis factor.

**Table 3:** The top 10 up- and downregulated differential expression genes from the 87 common genes for the different doses and exposure times in the in vivo and in vitro guinea pig data. FC, fold change.

Gene (Down)	log_2_ (FC)	Gene (Up)	log_2_ (FC)
Slc7a13	−1.98	Gsta2	7.07
Cbr1	−1.73	Gsta5	7.07
LOC100360601	−1.73	Mt2A	6.47
LOC102556347	−1.73	Mt1m	6.47
Ppp1r3c	−1.42	Gsto1	3.04
Cltrn	−1.41	Mt1	2.69
Smoc1	−1.17	Uchl1	2.51
Cd200	−1.15	Gstp1	1.65
Col11a1	−1.04	Rrm2	1.61
Bend5	−1.00	Aldh1a7	1.57

**Table 4:** Activation scores for kidney injury modules based on gene expression data collected at different doses and exposure times during the in vivo and in vitro guinea pig studies. Bold font indicates values above the 2.0 significance threshold. HD, high dose; LD, low dose.

Kidney Injury Modules	In Vivo				In Vitro			
	9 h		33 h		12 h		24 h	
	LD	HD	LD	HD	LD	HD	LD	HD
Dilatation	1.51	**2.72**	**5.65**	**10.45**	0.51	**2.94**	0.63	**2.59**
Hyaline cast	**2.38**	**3.54**	**4.29**	**9.64**	0.32	1.73	0.41	1.20
Degeneration	**2.55**	**3.09**	**4.74**	**7.58**	**2.74**	1.10	0.31	0.96
Necrosis	**5.05**	**6.10**	**3.45**	**6.70**	1.57	**2.74**	1.77	1.72
Fibrogenesis	**2.07**	**2.79**	**2.18**	**3.67**	**3.03**	**5.11**	1.03	**2.34**
Intracytoplasmic inclusion body	0.68	1.43	0.17	**3.50**	1.51	**4.88**	0.39	0.85
Hypertrophy	0.32	−0.43	−0.04	1.50	−0.21	0.80	0.65	−0.52
Cellular infiltration	−1.63	−1.04	**2.15**	0.04	**4.06**	**2.05**	1.35	0.82

## Data Availability

The data presented in this study are available in NCBI’s GEO database for gene repository under accession number GSE226313.

## Supplementary Materials

The following supporting information can be downloaded at: https://www.mdpi.com/article/10.3390/ijms24087434/s1.
